# Temporary postoperative treatment with compartment-unloading knee braces or wedge insoles does not improve clinical outcome after partial meniscectomy

**DOI:** 10.1007/s00167-018-5106-0

**Published:** 2018-08-22

**Authors:** Dietmar Dammerer, Florian Fischer, Raul Mayr, Johannes Giesinger, Rene El Attal, Michael C. Liebensteiner

**Affiliations:** 1 Department of Orthopaedics, Medical University of Innsbruck, Anichstraße 35, 6020 Innsbruck, Austria; 2 Department of Trauma Surgery, Medical University of Innsbruck, Anichstr. 35, 6020 Innsbruck, Austria; 3 Innsbruck Institute of Patient-centered Outcome Research (IIPCOR), Dr.Stumpf Straße 56, 6020 Innsbruck, Austria; 4 Department of Trauma Surgery, Feldkirch Academic Hospital, Carinagasse 47, 6807 Feldkirch, Austria

**Keywords:** Partial meniscectomy, Knee brace, Wedge insole, Unloading therapy, Knee arthroscopy

## Abstract

**Purpose:**

To investigate whether temporary postoperative compartment-unloading therapy after arthroscopic partial meniscectomy (APM)—with either knee braces or wedge insoles—leads to superior clinical outcome as compared to controls. This difference in clinical outcome was tested in the form of two knee scores, physical activity and general health outcome over the first postoperative year.

**Methods:**

Sixty-three patients who underwent arthroscopic partial meniscectomy (APM) were randomized to one of the following three groups: 12 weeks postoperative knee compartment-unloading therapy with either a knee brace (brace group) or wedge insoles (insole group) or no specific postoperative therapy (control group). Patient-reported outcome was assessed with the International Knee Documentation Committee Subjective Knee Evaluation Form (IKDC Score), the Knee Injury and Osteoarthritis Outcome Score (KOOS), the MARX score (physical activity) and the SF-12 (general health).

**Results:**

Sixty-three patients were available for analysis. Except for the SF-12 mental score, all other scores showed significant improvement over time. With regard to the hypotheses proposed, no significant group * time interactions were observed for any of the outcome parameters. This means that the group (i.e. the type of postoperative treatment) was not related to the degree of improvement of any of the scores.

**Conclusions:**

It was concluded that 12 weeks of compartment-unloading therapy—with either a knee brace or wedge insoles—is ineffective with regard to clinical outcome after APM. This applies to the knee score outcome, physical activity and general health outcome over the first year following APM.

**Level of evidence:**

Randomized controlled trial, Level I.

## Introduction

Previous studies reported that following medial arthroscopic partial meniscectomy (APM) patients demonstrated increased varus loading (knee adduction moment; KAM) in the meniscectomised knee as compared to the controls and partly also as compared to the contralateral side [5, 9, 36, 37]. This effect was attributed to the loss of meniscal volume.

Increased KAM was found to enlarge the pressure on the medial compartment of the knee and to play a key role in the development of degenerative joint disease [1, 2, 4, 9, 20, 26, 32, 34, 37, 39]. Increased KAM was associated with incidence, severity and progression of medial tibiofemoral osteoarthritis [5, 36]. Increased KAM may also play a key role in the etiology of the so-called post-arthroscopic bone marrow edema that seems to have an incidence between 7 and 34% APM [18, 23, 31, 33]. In synopsis, the above-mentioned knowledge of increased KAM after medial APM and the implications of KAM for degenerative joint disease have led to a shift from rather liberal to a now more cautious surgical indication for APM.

In addition, it might also be speculated whether potential postoperative compartment-unloading tactics would be beneficial for the outcome of APM. Compartment unloading can be achieved with knee braces [3, 6, 7, 10, 16, 19, 21] and wedge insoles [6, 16, 17, 24, 27]. These studies tested the unloading effects in patients with unicompartmental knee osteoarthritis with or without frontal plane knee malalignment. Interestingly, others expanded the field and verified a compartment-unloading effect of knee braces also in healthy subjects with normally aligned knees [25]. The authors suggested that such braces be potentially used to unload a knee compartment after cartilage repair procedures. However, no previous studies had applied these ideas to the issue of APM at the time this study was initiated.

Therefore, it was the aim of this study to investigate whether temporary postoperative compartment-unloading therapy after APM—with either knee braces or wedge insoles—would lead to superior clinical outcome as compared to controls. It was hypothesized that a difference in clinical outcome in terms of two knee scores (Hypotheses 1 and 2), physical activity (Hypothesis 3) and general health outcome (Hypothesis 4) over the first postoperative year will be shown.

## Materials and methods

### A randomized-controlled study design was applied

Patients who underwent arthroscopic partial meniscectomy (APM) as part of the clinical routine at our medical university hospital were considered for inclusion. In the case of degenerative meniscus lesions surgical indication was made conservatively (no success with conservative therapy, giving way, blocked knee joint).

Exclusion criteria were: (1) scheduled combined or staged surgical procedures (e.g. osteotomy or ligament reconstruction, (2) scheduled bicompartmental meniscectomy, (3) full-thickness chondral lesions as determined with preoperative MRI (4) rheumatoid arthritis or (5) osteoarthritis (Kellgren–Lawrence Grade other than 0 or 1). The concept of unloading a recently partly resected medial meniscus was regarded as appropriate only for the case of a neutral or slightly varus leg axis. The concept of unloading a recently partly resected lateral meniscus was regarded as appropriate only for the case of a neutral or slightly valgus leg axis. Therefore, the patient’s mechanical tibiofemoral angle (mTFA) was taken from a weight-bearing whole leg radiograph during bipedal stance at the time of enrollment. The mTFA was measured as suggested by previous researchers [11, 15, 35]. The center of the femoral head was connected to the center of the distal femur to define the mechanical axis of the femur. The mechanical axis of the tibia was defined as a line from the center of the proximal tibia to the center of the ankle joint. The angle between the mechanical axis of the femur and the mechanical axis of the tibia was taken as mTFA [11]. The angle was determined to one decimal place. The applied method was reported as being accurate and reliable in previous research [35]. A knee with a mTFA from 2.9° varus to 2.9° valgus was defined as neutrally aligned. Knees with a varus or valgus mTFA in excess thereof were defined as varus and valgus knees, respectively. Then the additional exclusion criteria (6) scheduled medial APM in a valgus knee and (7) scheduled lateral APM in a varus knee were applied.

APM was conducted as part of the clinical routine, either as day surgery or as part of a short hospital stay. Tourniquets were used depending on the surgeon’s preference. Great attention was paid to maintaining a stable peripheral rim during APM. In none of the procedures was the rim fully interrupted/resected. Complete interruption of the peripheral rim is considered to be complete meniscus loss from a functional standpoint. All procedures were conducted by experienced orthopaedic consultants or under their direct supervision. At the time of APM additional exclusion criteria were applied: (1) unanticipated full-thickness chondral lesions, (2) unanticipated need for additional procedures (e.g. chondral procedures), (3) APM performed in both compartments and (4) intraoperative change to other meniscus procedures (e.g. total meniscectomy, meniscus repair). Physical exercise started from the day of surgery. Neither active nor passive range of motion was restricted. Weight-bearing was restricted to 50 percent bodyweight for ten days. All patients were prescribed a series of physical therapy on an outpatient basis (8–10 sessions of 40 min each).

The intervention under investigation was the postoperative treatment after APM. Patients were randomized to either the brace group, the insole group or the control group. In the brace group, patients used an unloading knee brace “OA Nano” (DJO UK Ltd., Guildford, Surrey, UK). The brace applied a valgus force in patients who had undergone medial APM and a varus force in patients who had undergone lateral APM. The above-mentioned brace is designed for light to moderate knee OA and provides unloading with a lightweight magnesium frame. This was done to unload the joint compartment in which the meniscus had just been partly resected. The knee brace was worn by the patients for a minimum of 5 h a day over a period of 12 weeks as protocolled in an individual diary. In the insole group, patients received a custom-made wedge insole of 5 millimeters. To achieve postoperative compartment unloading patients who had undergone medial APM received a lateral wedge insole. Patients who had undergone lateral partial meniscectomy received a medial wedge insole. Again, insoles were worn for a minimum of 5 h a day over a period of 12 weeks. To facilitate patients’ adherence to the treatment protocol they were given insoles for both outdoor and indoor shoes. Patients in the control group did not receive any specific additional compartment-unloading therapy.

Based on previous recommendations [41] the clinical outcome was assessed with patient-reported outcome measures (PROMs) in a threefold manner: (a) knee-specific outcome, (b) physical activity and (c) general health. For the knee-specific outcome the International Knee Documentation Committee Subjective Knee Evaluation Form (IKDC Score) was applied [13]. Validity, reliability and responsiveness were demonstrated for many knee disorders as well as meniscal pathologies [12, 14]. The 18 items are summed up for the raw score, which is then transferred to a 0–100 scale (0: worst, 100: best). The Knee Injury and Osteoarthritis Outcome Score (KOOS) [29] was used as a second knee-specific outcome instrument. The KOOS consists of the five subscales pain (9 items), symptoms (7 items), activities of daily living (17 items), sport and recreation function (5 items), and knee-related quality of life (4 items). Each scale is summed up and transferred to a 0–100 scale (0: worst, 100: best). Also the KOOS was proven to be an appropriate instrument for determining the outcome of meniscal surgery [28, 30]. Physical activity was assessed with the MARX score, which determined activity in terms of running, cutting, decelerating and pivoting [22]. In each category points were given depending on frequency (0–4 points), which gave a total scale of 0–16 (0: worst, 16: best). General health was determined with the Short-form 12 (SF-12) that provides results in the form of a physical score and a mental score [40]. All above-mentioned outcome parameters were collected preoperatively, and postoperatively at 6 weeks, 3 months, 6 months and 12 months.

The Ethics Committee of the Medical University of Innsbruck approved the study protocol (No. AN-2014-0004 333/4.6). The study was registered at clinicaltrial.gov (Identifier: NCT02190188).

### Statistical analysis

Sample characteristics are given as means and standard deviations as well as absolute and relative frequencies. Analysis of the impact of the intervention was performed with the help of linear mixed models. The models included the outcome parameter (IKDC, KOOS, MARX, SF-12) as dependent variable, time point and study group as fixed effects, the two-way interaction of group and time, and a diagonal covariance structure. Separate models were run for the various outcome parameters. In such a model the impact of the intervention is reflected by the group-by-time interaction term. P values below 0.05 were considered to be statistically significant. All statistical analyses were performed with SPSS 21.0 (IBM, Chicago, IL, USA).

The power analysis for the study was done for the main hypotheses and considered the two-way interaction of group-by-time (three groups, five time points) in a repeated measure analysis of variance that is a group difference in parameter change over time. In such an analysis, a difference in change over time with an effect size of *f* = 0.14 can be detected with power = 0.80 (*α* = 0.05, correlation among repeated measures is 0.70 and non-sphericity correction is 1.0) if the sample size is *N* = 16 per group (*N* = 48 in total). To account for an attrition rate of 30% we aimed to include *N* = 63 at baseline. Power analysis was done with the software G*Power 3.1.9.2 [8].

## Results

**Fig. 1:**
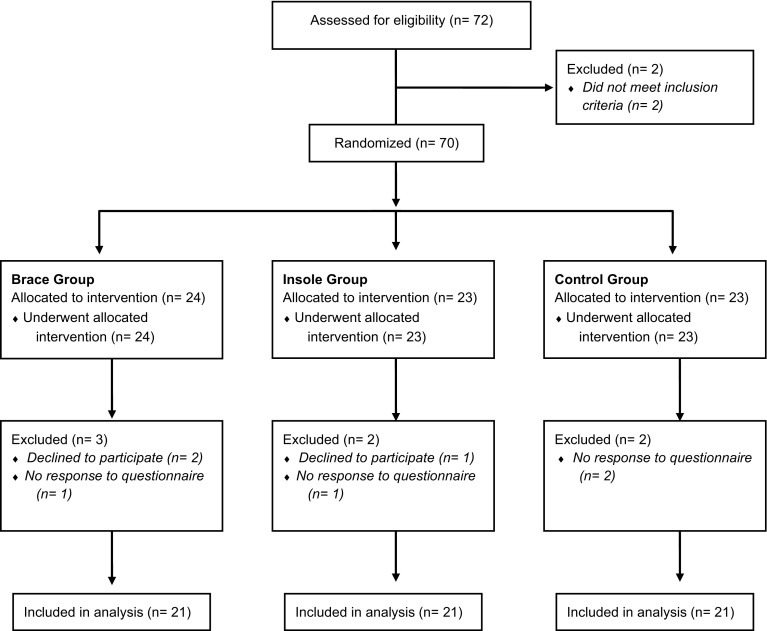
Flow chart illustrating patients involved in the study

Sixty-three patients were available for analysis. The flow of patients is provided in Fig. 1. Detailed patient characteristics for the groups ‘brace’, ‘insole’ and ‘controls’ are provided in Table 1.

**Table 1:** Patient demographics

	Brace	Insole	Control
Sex			
Female	10	10	10
Male	11	11	11
Age (years)	50.6 (± 11.6)	53.3 (± 11.2)	48.0 (± 13.5)
Body Mass Index	24.7 (± 3.6)	26.5 (± 7.0)	26.1 (± 11.1)
Compartment			
Medial	19 (90.5%)	19 (90.5%)	18 (85.7%)
Lateral	2 (9.5%)	2 (9.5%)	3 (14.3%)

Regarding knee-specific outcome, the IKDC score significantly improved in all groups over time (*p* < 0.001). Neither were there any significant differences between groups (n.s.), nor were there any significant group * time interactions (n.s., Hypothesis 1, Tables 2, 3).

**Table 2:** Descriptive statistics for the patient-reported outcome parameters (means ± standard deviations)

	SF-12 physical	SF-12 mental	IKDC	MARX	KOOS-S	KOOS-P	KOOS-ADL	KOOS-SP	KOOS-QOL
Pre									
Brace	38.7 ± 10.5	54.8 ± 6.8	49.6 ± 16.7	2.2 ± 4.0	64.1 ± 20.4	58.6 ± 22.8	68.9 ± 18.1	39.8 ± 23.9	37.2 ± 23.3
Insole	39.3 ± 9.4	51.5 ± 11.3	50.5 ± 14.4	1.5 ± 2.4	65.4 ± 17.5	59.9 ± 17.7	68.9 ± 19.7	40.3 ± 25.1	35.9 ± 12.6
Control	34.8 ± 8.5	52.7 ± 10.9	45.8 ± 10.4	2.4 ± 4.1	54.9 ± 14.8	47.9 ± 15.3	58.3 ± 18.4	25.7 ± 18.6	26.5 ± 13.4
6 weeks									
Brace	45.5 ± 9.1	52.8 ± 10.2	67.8 ± 18.3	1.8 ± 3.1	81.6 ± 14.2	80.0 ± 17.3	89.0 ± 13.5	66.5 ± 27.8	62.2 ± 24.0
Insole	40.0 ± 12.3	50.2 ± 10.4	64.4 ± 18.0	1.3 ± 2.4	78.4 ± 18.4	75.8 ± 18.6	78.8 ± 19.9	54.5 ± 27.9	58.8 ± 23.6
Control	42.5 ± 10.8	54.5 ± 10.2	65.0 ± 16.5	1.3 ± 2.1	72.8 ± 18.7	75.9 ± 17.7	84.1 ± 14.7	56.4 ± 28.2	55.4 ± 22.9
12 weeks									
Brace	50.0 ± 8.8	53.0 ± 9.8	76.7 ± 16.7	4.5 ± 5.3	82.7 ± 14.3	85.7 ± 17.6	92.2 ± 11.8	76.9 ± 22.1	74.7 ± 22.5
Insole	44.2 ± 10.8	50.5 ± 12.2	66.9 ± 21.9	2.2 ± 3.2	77.3 ± 20.2	77.5 ± 18.5	81.7 ± 21.6	61.5 ± 28.7	62.2 ± 27.8
Control	48.5 ± 9.0	56.5 ± 4.2	74.3 ± 16.9	3.6 ± 4.8	79.5 ± 16.9	79.1 ± 18.9	85.8 ± 19.4	68.2 ± 25.5	61.8 ± 23.7
6 months									
Brace	49.2 ± 8.0	55.9 ± 9.2	77.4 ± 15.6	2.4 ± 2.8	85.5 ± 12.8	89.6 ± 12.5	92.7 ± 10.8	80.5 ± 17.6	77.6 ± 22.4
Insole	48.3 ± 9.9	52.3 ± 9.6	73.4 ± 17.9	2.8 ± 2.2	84.5 ± 14.4	80.4 ± 19.4	85.8 ± 20.0	74.2 ± 28.5	72.6 ± 72.6
Control	46.5 ± 9.4	56.4 ± 6.7	69.2 ± 20.5	3.4 ± 4.0	80.5 ± 18.9	81.7 ± 17.7	86.2 ± 16.4	61.5 ± 27.3	59.9 ± 22.1
12 months									
Brace	50.6 ± 7.3	55.6 ± 8.1	77.9 ± 18.4	2.3 ± 4.3	89.1 ± 9.5	87.5 ± 11.9	92.9 ± 9.5	78.4 ± 20.4	72.3 ± 23.9
Insole	48.3 ± 10.2	55.4 ± 7.8	79.6 ± 19.0	3.3 ± 3.4	86.2 ± 16.3	86.7 ± 17.6	88.5 ± 21.1	74.0 ± 28.2	75.4 ± 28.0
Control	46.9 ± 11.5	53.1 ± 8.7	70.9 ± 19.2	3.0 ± 3.3	75.5 ± 22.6	77.4 ± 22.5	82.8 ± 21.5	60.0 ± 30.9	58.7 ± 26.3

*IKDC* international knee documentation committee subjective knee evaluation form, *KOOS-S* Knee Injury and Osteoarthritis Outcome Score—subscore SYMPTOMS, *KOOS-P* Knee Injury and Osteoarthritis Outcome Score—subscore PAIN, *KOOS-ADL* Knee Injury and Osteoarthritis Outcome Score—subscore ACTIVITIES OF DAILY LIVING, *KOOS-SP* Knee Injury and Osteoarthritis Outcome Score—subscore SPORT & RECREATION, *KOOS-QOL* Knee Injury and Osteoarthritis Outcome Score—Subscore QUALITY OF LIFE

**Table 3:** Inferential statistics for the patient-reported outcome parameters (linear mixed model)

*p* values	SF-12 physical	SF-12 mental	IKDC	MARX	KOOS-S	KOOS-P	KOOS-ADL	KOOS-SP	KOOS-QOL
Factor group	0.072	0.106	0.175	0.637	0.005	0.012	0.006	0.001	0.001
Factor time	< 0.001	0.602	< 0.001	0.014	< 0.001	< 0.001	< 0.001	< 0.001	< 0.001
Group * time interaction	0.656	0.727	0.761	0.818	0.843	0.720	0.674	0.601	0.863

*IKDC* international knee documentation committee subjective knee evaluation form, *KOOS-S* Knee Injury and Osteoarthritis Outcome Score—subscore SYMPTOMS, *KOOS-P* Knee Injury and Osteoarthritis Outcome Score—Subscore PAIN, *KOOS-ADL* Knee Injury and Osteoarthritis Outcome Score—Subscore ACTIVITIES OF DAILY LIVING, *KOOS-SP* Knee Injury and Osteoarthritis Outcome Score—Subscore SPORT & RECREATION, *KOOS-QOL* Knee Injury and Osteoarthritis Outcome Score—Subscore QUALITY OF LIFE

Regarding knee-specific outcome, all KOOS subscores significantly improved over time (*p* < 0.001). For all KOOS scores, the factor group had a significant effect (0.001 < *p* < 0.012) with patients in the control group showing lower values on all KOOS subscores. However, no significant group * time interactions were observed (n.s. < *p* < n.s.). This means that the group (i.e. the type of postoperative treatment) was not related to the *degree* of improvement of the KOOS scores (Hypothesis 2, Tables 2, 3).

The physical activity level as determined by the MARX score significantly increased over time in all groups (*p* = 0.014). Neither were there any significant differences between groups (n.s.), nor were any significant group * time interactions observed (n.s., Hypothesis 3, Tables 2, 3).

Health-related quality of life was assessed in terms of the SF-12 mental and physical scores. Whereas the SF-12 physical score significantly improved over time (*p* < 0.001), the mental score remained unchanged over time (n.s.). Neither of the two scores showed any significant effects for the factor group (n.s.). No significant group * time interactions were observed (n.s.), thus demonstrating that the group was not related to the degree of change in the SF-12 scores (Hypothesis 4, Tables 2, 3).

## Discussion

The main study finding was that the clinical outcome did not differ between patients using unloading braces or insoles or neither of the two in the first 12 weeks after APM. No statistically significant group * time effects were found with regard to knee-specific scores, physical activity scores or general health measures.

At the time this study was initiated there were no other published studies dealing with the question of postoperative compartment unloading following APM. Meanwhile, Thorning et al. also investigated this specific issue [38]. Those authors tested whether a valgus unloader knee brace could decrease KAM in patients following APM. Their 22 patients underwent gait analysis during the tasks walking, forward lunge and one-leg rise with and without the valgus brace. The authors reported that KAM did not significantly differ between trials with and without the brace, no matter which of the above-mentioned three tasks was concerned. Thorning et al. emphasized large inter-individual differences, with some of the subjects showing KAM reductions of 30% while others exhibited KAM increases of 20% as an effect of the valgus knee brace. It should be noted that the current study and the study by Thorning et al. investigated the same idea: compartment-unloading tactics after APM. However, several aspects restrict comparability. First and foremost, Thorning et al. conducted a cross-sectional study investigating biomechanical outcome parameters while our study project aimed at exploring patient-reported outcome parameters in a longitudinal study design. In addition, our study expanded the field beyond knee braces to also include wedge insoles as a potential tactic for unloading a knee compartment. Otherwise, the sample characteristics were comparable between the two studies with regard to age, but slightly different with regard to sex distribution (1:1 in our study, 1:2 in the study by Thorning et al.). What is different is that Thorning et al. focused on medial APM only. We tried to also include patients who had undergone lateral APM and treated them with varus braces and insoles. Despite all the aspects that restrict comparability between the two studies, both studies are congruent in that neither showed a positive effect of compartment-unloading devices, whether on KAM or on clinical score outcome. The investigation by Thorning et al. should be accorded great attention, as it was the first publication to deal with the subject of compartment unloading following APM. To our best knowledge, no other previous researchers investigated the issue of postoperative compartment unloading following APM.

Slightly different ideas were put forth by Orishimo et al. [25] who speculated whether medial compartment cartilage repair procedures should be treated postoperatively with compartment-unloading knee braces. To test their concept the authors applied valgus knee braces to 12 healthy individuals with neutrally aligned knees during gait analysis. They reported that the knee brace was able to significantly reduce KAM during walking without altering walking speed or sagittal knee kinematics. The findings of that study are very interesting as they demonstrate that the concept of compartment unloading also works in neutrally aligned knees. However, this study also demonstrated that their findings cannot be directly extrapolated to patients after cartilage repair. The findings obtained from the basic research by Orishimo et al. should first be tested in a longitudinal comparative clinical investigation similar to that done in our study for patients with APM.

Apart from the proposed hypotheses (differences between groups) it was observed that all groups improved over time. This is regarded as a positive effect of APM. However, positive outcome in the case of a restrictive surgical indication for APM is well known and was, therefore, not linked to a hypothesis.

The following limitations of our study should be acknowledged. First, the postoperative unloading treatment was applied for 12 weeks only. It might be speculated whether a longer intervention period would have led to an effect on clinical outcome. However, it is believed that 12 weeks is already long, but acceptable for the patient. It is assumed that longer periods of unloading treatment would lead to non-compliance in daily clinical practice and are, therefore, regarded as impracticable. Second, our participants unfortunately had inter-group differences in baseline KOOS values. Third, we relied solely on patient-reported outcome parameters. Additional imaging criteria (e.g. joint space narrowing taken from Rosenberg radiographs over the first postoperative year) would also have been of great interest. In addition, the study might have suffered from the fact that we had both medial and lateral APM in all groups. It would have been advantageous to have more homogeneous populations of purely medial APM in all groups.

Despite its weaknesses, the study significantly contributes to scientific knowledge, as it is the second study to investigate the issue of compartment-unloading treatments following APM and the first to investigate clinical outcome in a longitudinal prospective, comparative design.

The study findings are regarded as clinically relevant because they show that there is currently no support for the theoretical concept of postoperative compartment unloading treatment (either insole or brace) after APM.

## Conclusions

It was concluded that 12 weeks of compartment-unloading therapy—with either a knee brace or wedge insoles—is ineffective with regard to clinical outcome following APM. This applies to the knee score outcome, physical activity and general health outcome over the first year after APM.
